# Incidental MRI findings of left ventricular myocardial scar in atrial fibrillation patients is associated with increased stroke risk

**DOI:** 10.1186/1532-429X-16-S1-P92

**Published:** 2014-01-16

**Authors:** Promporn Suksaranjit, Krishna Velagapudi, Kavitha Damal, Lowell Chang, Allen Rassa, Erik T Bieging, Nassir F Marrouche, Brent D Wilson, Christopher J McGann

**Affiliations:** 1 University of Utah, Salt Lake City, Utah, USA

## Background

Left ventricular late gadolinium enhancement (LV-LGE) is a known incidental cardiac magnetic resonance (CMR) finding in atrial fibrillation (AF) patients and has recently been associated with increased mortality [1]. While LV-LGE is an indicator of poor prognosis in patients with cardiomyopathy, data on the clinical significance of incidental findings of LV-LGE in AF patients is still unavailable. We aimed to evaluate the association of this incidental finding to ischemic stroke in atrial fibrillation.

## Methods

A 1:2 observational case-control study was conducted with a dataset of cases with new onset ischemic stroke after 1st CMR scan and controls matched by age and sex. Cases and controls were selected from a database of 762 AF patients without any prior history of MI who underwent cardiac MRI exams between June 2006 and January 2013. We manually reviewed the electronic medical records of all patients with CMR and sequentially selected the first identified age and sex matched controls (subjects without stroke event after their CMR scan). Two experienced readers scrutinized the CMR scans of cases and controls for presence of left ventricular delayed enhancement. We then collected all comorbidities and conducted univariate (Chi-square test/t-test as appropriate) and multivariate analyses to examine the association between LV-LGE and occurrence of stroke.

## Results

Out of 762 patients, we identified 14 cases (1.8%) with new onset ischemic stroke after the first MRI scan. Of these cases, 2 (14%) were found to have LV-LGE. We age and sex matched these 14 cases with 28 controls in a 1:2 fashion. Description of demographic variables and univariate measures of association are presented in Table 1. Prior stroke and use of calcium channel and beta blockers were the only statistically significant differences between the two groups. We found a statistically significant association between LV-LGE and occurrence of stroke (McNemar's Chi square test, P = 0.0034). In a conditional logistic regression model, LV-LGE predicted the occurrence of stroke perfectly. After accounting for LV-LGE, CHADS2 score was the only variable that predicted the occurrence of stroke in AF patients (odds ratio = 2.05, 95% CI = 1.01 - 4.16, P = 0.045). The presence of LV-LGE incrementally predicts stroke risk with higher CHADS2 score Table 2.

**Table 1:** Demographics

	Ischemic stroke after CMR scan	No stroke	P value
Sex	6 females	12 females	1.00

Age	67.47 ± 12.16	67.50 ± 9.00	0.99

DM	2	3	0.7

HTN	8	17	0.8

CHF	3	3	0.35

Prior Stroke	4	1	0.018

Dyslipidemia	6	6	0.147

Obstructive sleep apnea	1	4	0.5

Thyroid disease	3	7	0.79

Smoking history	4	7	0.804

Class I antiarrythmic drug	2	2	0.45

Class III antiarrythmic drug	2	4	1.00

Anticoagulants	9	12	0.19

ASA	3	9	0.46

Statins	5	6	0.32

Calcium channel blocker	1	12	0.018

Beta blocker	9	7	0.013

ACEI/ARBs	7	12	0.661

Diuretics	7	7	0.105

GFR	72.64 ± 21.04	75.28 ± 23.12	0.72

LV end diastolic diameter	4.87 ± 1.03	4.93 ± 0.69	0.82

LA area	29.67 ± 1.66	30.78 ± 7.55	0.636

BMI	27.22 ± 7.82	30.79 ± 6.12	0.112

**Table 2:** Delta Method margins show LV-LGE as an incremental predictor of stroke after first CMR scan in AF patients based on CHADS2 score

CHADS2 Score	Margin	Standard Error	P value	95% CI
0	0.111	0.105	0.289	-0.094-0.316

1	0.313	0.116	0.007	0.085-0.540

2	0.400	0.155	0.010	0.096-0.704

3	0.571	0.187	0.002	0.205-0.938

## Conclusions

In this case-control study of AF patients without a history of MI, an incidental finding of LV-LGE is shown to predict the risk of stroke, especially in the case of patients with a higher CHADS2 score.

## Funding

Not applicable.
